# Supplementary material for: EDF1 coordinates cellular responses to ribosome collisions
Source: eLife. 2020 Aug 3;9:e58828. doi: 10.7554/eLife.58828 (PMC7486125; doi:10.7554/eLife.58828)
Supplement: Figure 3—source data 1. [file elife-58828-fig3-data1.docx]

**Figure 3 – Source Data 1**

|  | **EDF1-ribosome** | **Mbf1-ribosome** |
| --- | --- | --- |
| Ribosomal state | Post State | Rotated state |
| Microscope | FEI Titan Krios | FEI Titan Krios |
| Camera | K2 | K2 |
| Voltage (kV) | 300 | 300 |
| Pixel size (Å) | 1.059 | 1.059 |
| Electron dose (e-/Å^2^) | 42 | 42 |
| Defocus range (µm) | 0.5 - 2.5 | 0.5 - 2.5 |
| Particles after 2D (no.) | 95832 | 398371 |
| Final particles (no.) | 81976 | 57350 |
| **Model Composition** |  |  |
| Protein residues | 5074 | 4918 |
| RNA bases | 1815 | 1946 |
| **Resolution (Å)** | 2.9 | 3.0 |
| FSC threshold | 0.143 | 0.143 |
| Map CC | 0.88 | 0.84 |
| Map sharpening B-factor (Å^2^) | -20 | -30 |
| **RMS Deviations** |  |  |
| Bond lengths (Å) | 0.008 | 0.007 |
| Bond angles (°) | 0.945 | 0.969 |
| **Validation** |  |  |
| MolProbity score | 1.67 | 1.69 |
| Clashscore | 5.79 | 5.92 |
| Poor rotamers (%) | 0.00 | 0.02 |
| **Ramachandran Plot** |  |  |
| Disallowed (%) | 0.18 | 0.21 |
| Allowed (%) | 4.88 | 5.16 |
| Favored (%) | 94.94 | 94.64 |
|  |  |  |
