# Supplementary material for: EDF1 coordinates cellular responses to ribosome collisions
Source: eLife. 2020 Aug 3;9:e58828. doi: 10.7554/eLife.58828 (PMC7486125; doi:10.7554/eLife.58828)
Supplement: Transparent reporting form [file elife-58828-transrepform.docx]

***eLife’s* transparent reporting form**

We encourage authors to provide detailed information *within their submission* to facilitate the interpretation and replication of experiments. Authors can upload supporting documentation to indicate the use of appropriate reporting guidelines for health-related research (see [EQUATOR Network](http://www.equator-network.org/%20)), life science research (see the [BioSharing Information Resource](https://biosharing.org/)), or the [ARRIVE guidelines](http://www.plosbiology.org/article/info:doi/10.1371/journal.pbio.1000412) for reporting work involving animal research. Where applicable, authors should refer to any relevant reporting standards documents in this form.

If you have any questions, please consult our Journal Policies and/or contact us: [editorial@elifesciences.org](mailto:editorial@elifesciences.org).

**Sample-size estimation**

- You should state whether an appropriate sample size was computed when the study was being designed
- You should state the statistical method of sample size computation and any required assumptions
- If no explicit power analysis was used, you should describe how you decided what sample (replicate) size (number) to use

Please outline where this information can be found within the submission (e.g., sections or figure legends), or explain why this information doesn’t apply to your submission:

No sample-size calculation was performed. Sample size was determined to be adequate based on the magnitude and consistency of measurable differences between groups and low observed variability between samples.

**Replicates**

- You should report how often each experiment was performed
- You should include a definition of biological versus technical replication
- The data obtained should be provided and sufficient information should be provided to indicate the number of independent biological and/or technical replicates
- If you encountered any outliers, you should describe how these were handled
- Criteria for exclusion/inclusion of data should be clearly stated
- High-throughput sequence data should be uploaded before submission, with a private link for reviewers provided (these are available from both GEO and ArrayExpress)

Please outline where this information can be found within the submission (e.g., sections or figure legends), or explain why this information doesn’t apply to your submission:

All experiments were replicated and all attempts at replication were successful and consistent. For proteomics experiments replicates clustered together in PCA, and we observed low coefficient of variation among replicates.

Additionally, for proteomics experiments, we chose n=3 or n=4 independent biological replicate given the limitation of the available TMT channels (information available in each corresponding figure legends and when available in individual experimental schemes in corresponding figure panel). No data were excluded from the analyses. Proteomics samples for comparison with TMT and TMTpro reagents, were randomly allocated in the TMT/TMTpro group and replicates were in adjacent channels. For other experiments, no randomization was done.

Ubiquitin remnant profiling and proximity labeling assays were performed in biological triplicate (n=3) for each condition tested. PCA was used to determine sample clustering, and low coefficient of variance was observed between biological replicates.

Stalling reporter assays (combined with flow cytometry) were performed in biological triplicate (n=3); for each replicate single-cell fluorescence intensities for 10,000 individual events were measured. Mean +/- standard deviation for n=3 reported for each condition tested; ROUT method (Q=10%) was used to identify outliers (information available in figure legends and methods section). No data were excluded from the analyses.

Luciferase assays: Renilla (RLuc) and firefly (Fluc) Luciferase values for three technical replicate wells were averaged to compute Fluc:RLuc ratio for an individual biological replicate. Five or six biological replicates for each experimental condition were measured, with the mean and SD reported (information available in figure legend and methods section). No data were excluded from the analyses.

Analytical sucrose gradient fractionation followed by immunoblotting was performed at-least twice. The number of replicates for each condition tested is indicated in the figure legend. Quantitative sucrose gradient fractionation for polysome proteomics were performed between n=3 to n=4 as indicated above. Information available in figure legends, main text, and materials and methods.

**Statistical reporting**

- Statistical analysis methods should be described and justified
- Raw data should be presented in figures whenever informative to do so (typically when N per group is less than 10)
- For each experiment, you should identify the statistical tests used, exact values of N, definitions of center, methods of multiple test correction, and dispersion and precision measures (e.g., mean, median, SD, SEM, confidence intervals; and, for the major substantive results, a measure of effect size (e.g., Pearson's r, Cohen's d)
- Report exact p-values wherever possible alongside the summary statistics and 95% confidence intervals. These should be reported for all key questions and not only when the p-value is less than 0.05.

Please outline where this information can be found within the submission (e.g., sections or figure legends), or explain why this information doesn’t apply to your submission:

This information when applicable can be found in all the relevant individual figure legends, data analyses portion of the methods sections, and supplementary tables (Source data tables) for the large proteomics dataset (individual p-values). Similarly, the information when applicable (# replicates, statistical tests used, multiplicity-adjusted p-values) is reported for flow-cytometric assays in the figure legends and described in the methods section.

(For large datasets, or papers with a very large number of statistical tests, you may upload a single table file with tests, Ns, etc., with reference to sections in the manuscript.)

**Group allocation**

- Indicate how samples were allocated into experimental groups (in the case of clinical studies, please specify allocation to treatment method); if randomization was used, please also state if restricted randomization was applied
- Indicate if masking was used during group allocation, data collection and/or data analysis

Please outline where this information can be found within the submission (e.g., sections or figure legends), or explain why this information doesn’t apply to your submission:

Each sample were allocated into experimental groups in a random manner and no restriction was applied. No masking was used.

**Additional data files (“source data”)**

- We encourage you to upload relevant additional data files, such as numerical data that are represented as a graph in a figure, or as a summary table
- Where provided, these should be in the most useful format, and they can be uploaded as “Source data” files linked to a main figure or table
- Include model definition files including the full list of parameters used
- Include code used for data analysis (e.g., R, MatLab)
- Avoid stating that data files are “available upon request”

Please indicate the figures or tables for which source data files have been provided:

All raw mass spectrometry data has been deposited in MassIVE repository, as indicated in the manuscript’s Data and materials availability section. Source data for all proteomics-based plots are provided in Source data tables (Figure 1-source data 1, Figure 2-source data 1, Figure 2-source data 2, Figure 5-source data 1, Figure 5-source data 2 and Figure 6-source data 1). Raw sequencing data were deposited in the GEO database under the accession number GSE149565; secure token for reviewers: uzajoeeultgrpsr. The cryo-EM structures reported here have been deposited in the Protein Data Bank under the accession codes 6ZVH (EDF1•ribosome) and 6ZVI (Mbf1•ribosome), and in the Electron Microscopy Data Bank under the accession codes EMD-11456 (EDF1•ribosome) and EMD-11457 (Mbf1•ribosome). Cryo-EM data collection, refinement and validation statistics are reported in Figure 3-source data 1.
